# The Impact of COVID-19 Pandemic During Lockdown on the Veterinary Profession in Romania: A Questionnaire-Based Survey

**DOI:** 10.3389/fvets.2021.737914

**Published:** 2021-11-10

**Authors:** Alexandra Nicoleta Mureşan, Sorin Morariu, Radu Andrei Baisan, Ruxandra Costea, Cosmin Mureşan

**Affiliations:** ^1^Faculty of Veterinary Medicine, Department of Internal Medicine, University of Agricultural Sciences and Veterinary Medicine of Cluj-Napoca, Cluj-Napoca, Romania; ^2^Faculty of Veterinary Medicine, Department of Parasitology and Dermatology, Banat's University of Agricultural Sciences and Veterinary Medicine “Regele Mihai I al României”, Timişoara, Romania; ^3^Faculty of Veterinary Medicine, Cardiology and Röntgendiagnostic Unit, “Ion Ionescu de la Brad” Iasi University of Life Sciences, Iasi, Romania; ^4^Faculty of Veterinary Medicine, Department of Anesthesia, University of Agronomic Sciences and Veterinary Medicine, Bucharest, Romania; ^5^Faculty of Veterinary Medicine, Department of Surgery, Anesthesia and Intensive Care, University of Agricultural Sciences and Veterinary Medicine of Cluj-Napoca, Cluj-Napoca, Romania

**Keywords:** COVID-19, SARS-CoV-2, lockdown, veterinary medicine, Romania

## Abstract

The lockdown period in Romania lasted for 60 days and had the purpose of limiting the spread of SARS-CoV-2 virus outbreak and manage its consequences through emergency measures on many important areas of activity. This study aimed to gather, assess, analyze and disseminate relevant social, economic, and medical aspects on the impact of COVID-19 pandemic during lockdown on the veterinary profession in Romania. A survey was created using an online questionnaire platform, and disseminated. The survey was completed by a total of 409 individuals. A high number of respondents (71.64%; *n* = 293) felt exposed to medium or high risk of infection with SARS-CoV-2 at their workplace and many (56.97%; *n* = 233) felt that their professional environment was more stressful than usual during lockdown. Almost all respondents (89.73%; *n* = 367) declared the implementation of several control measures recommended by FECAVA and FVE (e.g., social distancing, wearing protective equipment, hand washing), but few mentioned the opportunity of remote work or visiting restrictions. Overall, the results show that the impact of lockdown lies directly on four main categories of importance on veterinarian professional's life—human resource, activity management, relationship between veterinarian and authorities, and continuing education.

## Introduction

In early 2020, WHO declared the coronavirus disease 2019 (COVID-19) a Public Health Emergency of International Concern and, as severe acute respiratory syndrome coronavirus 2 (SARS-CoV-2) rapidly spread globally, it became a pandemic, infecting over 102.1 million confirmed patients and leading to over 2.2 million deaths (data as received by WHO from national authorities, as of 31st January 2021) (1).

In Romania, a European country with 19,530,631 residents (2), since the first confirmed case of infection on February 26th, 2020 (3, 4), the COVID-19 pandemic continued to accumulate 712.561 confirmed positive cases, and 17.841 deaths (Data from the National Institute of Public Health, as of 24th January 2021) (5).

The lockdown period in Romania, officially declared by the Romanian President on the 16th of March, 2020 as state of emergency, lasted for a total of 60 days and had the purpose of preventing the spread of COVID-19 and manage its consequences (6, 7). It was materialized by applying emergency measures on several important areas of activity, such as public order, economic sector, health, employment and social protection, justice, foreign affairs, and others, and continued on the 18th of May with a state of alert (8) that is still ongoing (as of 25th of June 2021) although some relaxation measures such as elimination of curfew or opening up of non-essential businesses have been taken recently.

Speculations on the impact of COVID-19 pandemic on veterinary healthcare professionals worldwide (9) led us to investigate how Romanian veterinarians were affected from an economic, social as well as medical point of view and offer an analysis of all aspects influenced by this disease.

Therefore, the aim of the current study was to gather, assess, analyze and disseminate relevant aspects considered essential regarding the impact of COVID-19 pandemic during lockdown on the veterinary profession in Romania. We hypothesized that Romanian veterinarians were affected from a financial, personal, professional, and mental well-being point of view by the COVID-19 pandemic and lockdown measures.

## Materials and Methods

### Survey Design

To achieve the objective, an online survey was created by using a user-friendly, commercially available, cloud-based survey tool (SurveyMonkey, http://www.surveymonkey.com). The survey was based on a comprehensive questionnaire composed of a set of 39 questions and one comment box, written in Romanian language, and grouped into four categories—demographics and human resource (17 questions), activity management (14 questions), relationship between veterinarian and authorities (3 questions), and continuing education (5 questions). The survey was created from scratch, and no question was taken from the pre-designed survey templates by SurveyMonkey or COVID-19-related surveys disseminated in other countries. It was based on the author's interests as well as current recommendations for SARS-CoV-2 control measures for veterinarians by FECAVA (Federation of European Companion Animal Veterinary Association) and FVE (Federation of Veterinarians of Europe) (10). Several types of questions were used in the survey, such as multiple-choice questions with a single answer (33 questions) or multiple-answer (3 questions), Likert scale questions (3 questions) and one comment box destined for free-text remarks. All participants received the same questions.

The survey was disseminated to the target group solely at national level by posting on social media (veterinary specific groups), and by email through the official veterinary board. The respondents were provided with a link and a QR code, and accessing the questionnaire was possible through several devices such as smartphone, tablet, laptop and desktop computer.

The survey was anonymous to the data collectors and did not collect, store or process personal data. Participants consent for data collection was offered by accepting to take part in the study. Completing the questionnaire was done exclusively online and was estimated to take about 8 min. Survey status was monitored weekly and 3 additional reminders were sent on social media during a period of 55 days, between the 16th of July and 10th of September 2020 (after lockdown ended).

### Quality Control

For enhanced accuracy of the results (and avoiding bias), several questions (*n* = 2) were provided with an “other” answer and a comment field, at the end of the mentioned choices, where respondents were invited to write their particular answer. In addition, a comment box was added at the end of the questionnaire, allowing the respondents to provide further information/comments/feedback if considered necessary.

### Data Analysis

The results sourced from respondents were processed on the same platform (SurveyMonkey). Descriptive statistics (counts and percentages) were used to summarize and describe the acquired data.

## Results

### Demographics and Human Resource

A total of 409 individuals completed the survey, out of which 66.99% (*n* = 274) were veterinarians and 11.25% (*n* = 46) primary veterinarians (Romanian specific degree). The other respondents were: veterinary assistants/technicians (4.4%; *n* = 18), veterinary graduated clinic managers (11%; *n* = 45), and academic teaching staff (6.11%; *n* = 25) (1 skipped answer; 0.24%). Almost half (46.45%; *n* = 190) were owners/ managers of a veterinary clinic or company in the veterinary field, 42.05% (*n* = 172) of them were working as employees in veterinary industry, 5.62% (*n* = 23) veterinary teaching staff, and 5.13% (*n* = 21) employees in veterinary practice (3 skipped answers; 0.73%).

Many (42.54%; *n* = 174) of respondents worked in first opinion practice, while 16.14% (*n* = 66) in a private veterinary clinic, private veterinary hospital (3.18%; *n* = 13), university teaching hospital (6.6%; *n* = 27), veterinary pharmacy/pharmaceutical distribution company (3.42%; *n* = 14), divisional veterinarians (14.18%; *n* = 58), laboratory animal facility (0.73%; *n* = 3), food control/public health (1.47%; *n* = 6), or national state authority structure (including the National Sanitary Veterinary and Food Safety Authority) (5.87%; *n* = 24). Another 5.87% (*n* = 24) identified as “other” (research, veterinary laboratory, farm, veterinary medical college, medical device company, or unemployed).

In terms of experience, most (33.25%; *n* = 136) had been practicing for <5 years, 20.78% (*n* = 85) between 6 and 10 years, 19.07% (*n* = 78) between 11 and 20 years, and 26.89% (*n* = 110) had over 20 years of experience.

Staff size was mostly small, with 63.81% (*n* = 261) respondents working in environments of <5 employees, 16.14% (*n* = 66) between 5 and 10 employees, 7.09% (*n* = 29) between 10 and 20 employees, 12.47% (*n* = 51) over 20 employees (2 skipped answers; 0.49%).

The preferred mode of communication between staff at the workplace was short discussions with social distancing of at least 1.5 meters (81.91%; *n* = 335), while 20.05% (*n* = 82) preferred written messages, 25.43% (*n* = 104) chose electronic messaging, and 7.33% (*n* = 30) other (as before, zoom meetings, video call) (1 skipped answer; 0.24%).

Only 14.18% (*n* = 58) of respondents have witnessed or performed staff reductions in the structure where they worked during and immediately after the cessation of the state of emergency. Most had not (82.15%; *n* = 336) and the rest didn't know whether yes or no (3.67%; *n* = 15). Furlough was seen by 17.11% (*n* = 70) of respondents, whereas 77.02% (*n* = 315) did not or were unaware (5.87%; *n* = 24). Only 10.27% (*n* = 42) declared there were resignations within the structure where they worked after cessation of confinement, while 82.64% (*n* = 338) responded there were none, 5.87% (*n* = 24) did not know and it was not applicable in 1.22% (*n* = 5) of cases.

Revenues from own salaries increased in 5.62% (*n* = 23), decreased in 36.19% (*n* = 148), and remained the same in more than half of cases (57.95%; *n* = 237) (1 skipped answer; 0.24%).

**Figure 1:**
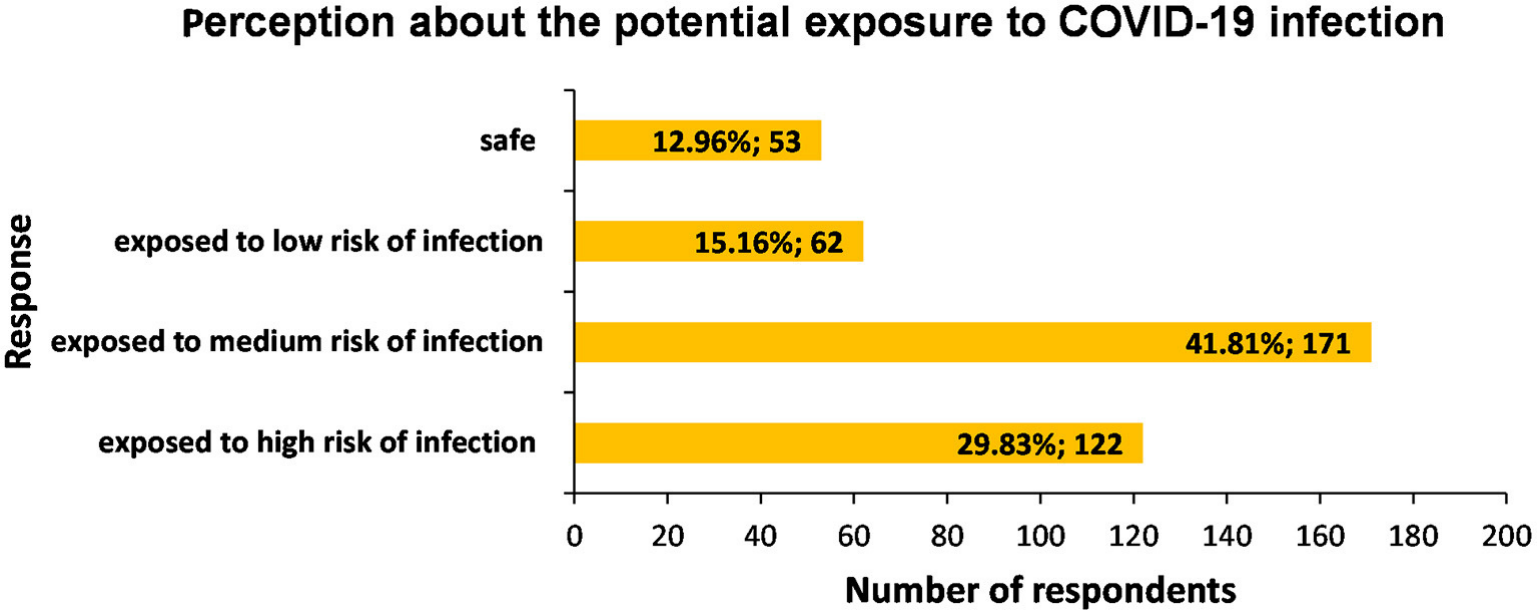
Distribution of respondents (*n* = 408) with respect to their perception about the potential exposure to COVID-19 infection at the workplace during confinement.

Only 12.96% (*n* = 53) of respondents felt safe at their workplace; 15.16% (*n* = 62) felt they were exposed to a low risk of infection, most felt exposed to medium risk of infection (41.81%; *n* = 171) and 29.83% (*n* = 122) felt exposed to a high risk of infection (1 skipped answer; 0.24%) (Figure 1).

Most respondents (56.97%; *n* = 233) agreed with the statement “During the state of emergency my professional activity was more stressful than usual”; 29.58% (*n* = 121) partially agreed with the statement, whereas 3.67% (*n* = 15) partially disagreed, 4.89% (*n* = 20) disagreed completely and 4.65% (*n* = 19) couldn't tell one way or the other (1 skipped answer; 0.24%).

At the time of the questionnaire completion, only 15.4% (*n* = 63) knew at least one person who tested positive for SARS-CoV-2; 22.98% (*n* = 94) were familiar with more than one person, but most (61.37%; *n* = 251) did not know anyone (1 skipped answer; 0.24%). Few respondents (0.98%; *n* = 4) had a positive test themselves, 7.33% (*n* = 30) were tested negative and the vast majority (91.44%; *n* = 374) had never been tested at all (1 skipped answer; 0.24%). Only 0.73% (*n* = 3) were hospitalized following a positive test for SARS-CoV-2 (5 skipped answers; 1.22%).

To the best of the respondents' knowledge, there were no persons from vulnerable categories (pregnant women, chronically ill, staff over 65) in the work teams of 66.99% (*n* = 274) respondents, whereas 27.63% (*n* = 113) did have them, and 5.13% (*n* = 21) were not aware of their presence (1 skipped answer; 0.24%).

All respondents considered that during the state of emergency they had opportunities such as (multiple choice answers): more free time (41.08%; *n* = 168), learning new things (29.58%; *n* = 121), changes in habits or daily routine (54.28%; *n* = 222), establishing/ resuming social ties (10.02%; *n* = 41), social assistance actions (8.07%; *n* = 33), reduction of pollution or traffic (55.99%; *n* = 229), awareness of the role/ limits of the human being (48.9%; *n* = 200) (2 skipped answers; 0.49%).

### Activity Management

Working hours were reduced in more than half of the workplaces (63.57%; *n* = 260) and increased in only 3.42% (*n* = 14). For 24.94% (*n* = 102) remained unchanged, 7.09% (*n* = 29) closed temporarily and 0.73% (*n* = 3) of workplaces were permanently closed (1 skipped answer; 0.24%).

Caseload dropped in 42.05% (*n* = 172) of workplaces offering veterinary care. 20.29% (*n* = 83) have seen an increase in caseload and 23.23% (*n* = 95) maintained a similar caseload to pre-emergency state. 4.4% (*n* = 18) of people replied “I don't know” and 9.78% (*n* = 40) declared the question was not applicable (1 skipped answer; 0.24%).

Almost half (45.72%; *n* = 187) of respondents declared that there were no changes in the type of cases seen at their workplace during the state of emergency. 18.09% (*n* = 74) received only emergencies, 19.8% (*n* = 81) received only scheduled cases, 1.96% (*n* = 8) redirected all cases, while for 14.18% (*n* = 58) the question was not applicable (1 skipped answer; 0.24%).

Most respondents (60.39%; *n* = 247) considered that the restrictions imposed during confinement have influenced the owner's decision to present to a veterinary service, but 19.32% (*n* = 79) responded “no,” 12.71% (*n* = 52) couldn't tell, and 7.33% (*n* = 30) responded “not applicable” (1 skipped answer; 0.24%).

Among the measures recommended by FECAVA and FVE advice for companion animal practitioners during the COVID-19 outbreak to be implemented in veterinary workplaces during the emergency period (10), almost 9 in 10 respondents (89.73%; *n* = 367) mentioned the obligation to wear appropriate protective equipment by all staff but few (12.96%; *n* = 53) were allowed to perform their professional activity from home. Detailed answers are provided in the table below (Table 1).

**Table 1:** Distribution of responses (counts and percentages) of a total number of 409 individuals completing the survey with respect to the control measures implemented at the workplace during the emergency period according to FECAVA and FVE advice leaflet during the COVID-19 outbreak (10).

**Control measures**	**Percentage of respondents**	**Number of respondents**
Shortening the working hours	49.14%	201
Restricting clinical activity exclusively to emergencies, admitting only urgent cases	15.65%	64
Obligation to wear appropriate protective equipment by all staff	89.73%	367
Hand washing regularly and thoroughly after each contact/ interaction with the pet and the owner, respectively	70.42%	288
Maintaining a safe distance of 2 meters between people	83.13%	340
Scheduling of cases exclusively by telephone/on-line	28.12%	115
Verbal information of animal owners about the new protection measures	44.25%	181
Informing through printed documents, placed in plain sight of animal owners about the new protection measures	51.34%	210
Restricting access to only one healthy adult person to accompany the pet in the consultation room/ waiting room	54.28%	222
Cleaning and disinfecting regularly of areas touched by many people (e.g., Door handles)	81.42%	333
Providing hand disinfectant for clients and placed in sight in the access/exit area	86.80%	355
Dividing the team into two (or 3) groups, avoiding physical contact between them	23.96%	98
Allowing professional activity to be performed from home	12.96%	53
Removing all items that can be touched by people in the waiting room	26.16%	107
Elimination of visiting hours for hospitalized patients and of non-essential visits (e.g., Pharmaceutical representatives, etc.)	31.54%	129
Contactless payment	39.12%	160

*One or more options were selected. All survey participants (n = 409) answered this question*.

More than half of respondents (52.32%; *n* = 214) did not know of colleagues belonging to vulnerable categories (pregnant women, chronically ill, staff over 65 years), but 7.09% (*n* = 29) took special measures for them (such as telework, administrative work, reducing/modifying the work schedule, maternity leave, unemployment, technical unemployment, furlough, no physical contact with the clients). 22.74% (*n* = 93) did not take any type of precautions, and 16.63% (*n* = 68) did not know (5 skipped answers; 1.22%).

About a third of respondents (31.54%; *n* = 129) did establish alternative ways in the workplace to maintain social distancing (e.g., telemedicine, non-contact consultation, triage by telephone). 46.21% (*n* = 189) did not, 6.85% (*n* = 28) didn't know or couldn't say and for 14.67% (*n* = 60) this was not applicable (3 skipped answers; 0.73%).

**Figure 2:**
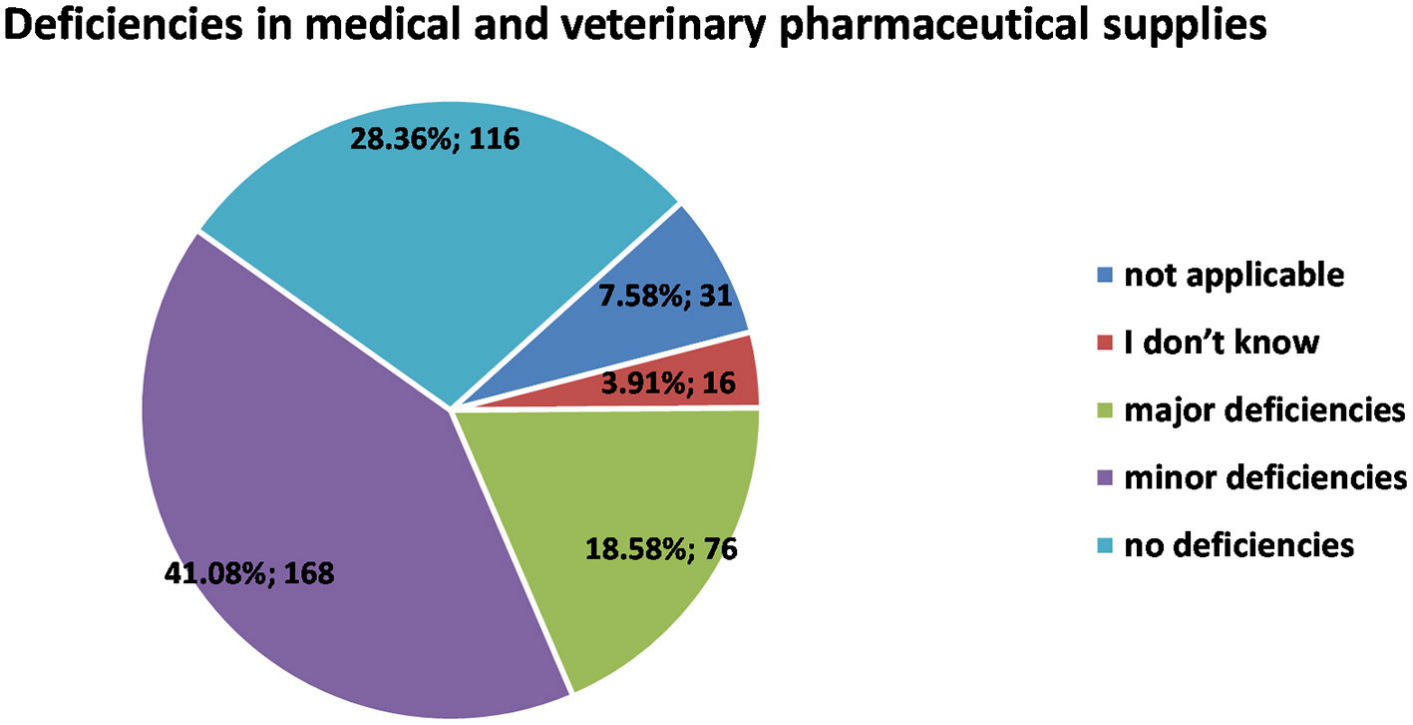
Distribution of respondents (*n* = 407) with respect to the encountered deficiencies in medical and veterinary pharmaceutical supplies at the workplace during confinement.

Most workplaces encountered deficiencies in the supply of veterinary pharmaceuticals and medical supplies during lockdown (Figure 2); 18.58% (*n* = 76) had major deficiencies, whereas minor deficiencies were experienced by 41.08% (*n* = 168). A quarter (28.36%; *n* = 116) did not have such deficiencies, 3.91% (*n* = 16) didn't know/ couldn't say, and for 7.58% (*n* = 31) the question was not applicable (2 skipped answers; 0.49%).

Only 11.25% (*n* = 46) received requests of support for pharmaceuticals, medical supplies, or medical devices (e.g., vital signs monitors, ventilators) from human healthcare providers during the state of emergency. 67.97% (*n* = 278) did not have any request, 8.56% (*n* = 35) didn't know or could not remember and for 11.49% (*n* = 47) the question was not applicable (3 skipped answers; 0.73%).

Most respondents (66.26%; *n* = 271) did not need to change the charging fees to maintain the professional activity in good conditions. A small percentage (4.65%; *n* = 19) decreased fees, some increased them (9.78%; *n* = 40) and for 18.83% (*n* = 77) the question was not applicable (2 skipped answers; 0.49%).

The majority of respondents (68.46%; *n* = 280) were asked by pet owners for information about the possibility of SARS-CoV-2 transmission from animals to humans or vice versa; 24.69% (*n* = 101) have not and 6.11% (*n* = 25) could not remember (3 skipped answers; 0.73%). On the other hand, very few (5.87%; 24) had been asked to consult an animal suspected of SARS-CoV-2, or to perform tests for such a diagnosis; 79.7% (*n* = 326) had not been asked and 2.2% (*n* = 9) could not remember; for 11.74% (*n* = 48) the question was not applicable (2 skipped answers; 0.49%).

While most respondents (68.95%; *n* = 282) had not been asked to consult an animal belonging to an owner suspected or confirmed positive for SARS-CoV-2, there were still 13.2% (*n* = 54) that received such request, 4.4% (*n* = 18) that could not remember, and the question was not applicable for 12.96% (*n* = 53) (2 skipped answers; 0.49%). Similar results were yielded by the question of whether respondents have been asked by a pet owner to vaccinate a clinically healthy pet with a commercial canine/feline vaccine against enteric coronavirus, for possible cross-protection against SARS-CoV-2. Most (72.86%; *n* = 298) were not, but there were still 9.05% (*n* = 37) that had been asked to perform this task, 2.44% (*n* = 10) couldn't remember and for 15.16% (*n* = 62) the question was not applicable (2 skipped answers; 0.49%).

### Relationship Between Veterinarian and Authorities

During the state of emergency, 17.85% (*n* = 73) of the respondents had to travel often (more than twice per week) in the field or at the client's home to provide veterinary care, 23.96% (*n* = 98) traveled occasionally (1–2 per week), but 17.36% (*n* = 71) were never asked. 22% (*n* = 90) refused to perform such services and 18.34% (*n* = 75) considered the question not applicable (2 skipped answers; 0.49%).

While traveling in the field or at the client's home, 13.94% (*n* = 57) have been stopped by the police more than once a week, and 22.98% (*n* = 94) only once a week to verify compliance with the prohibitions imposed during the state of emergency. 26.89% (*n* = 110) were never stopped and for 35.7% (*n* = 146) the question was not applicable (2 skipped answers; 0.49%).

Only 1.96% (*n* = 8) were fined while traveling in the field or at the client's home for professional purpose, whilst 61.61% (*n* = 252) did not. The question was not applicable for about a third of the respondents (35.7%; *n* = 146) (3 skipped answers; 0.73%).

### Continuing Education

**Figure 3:**
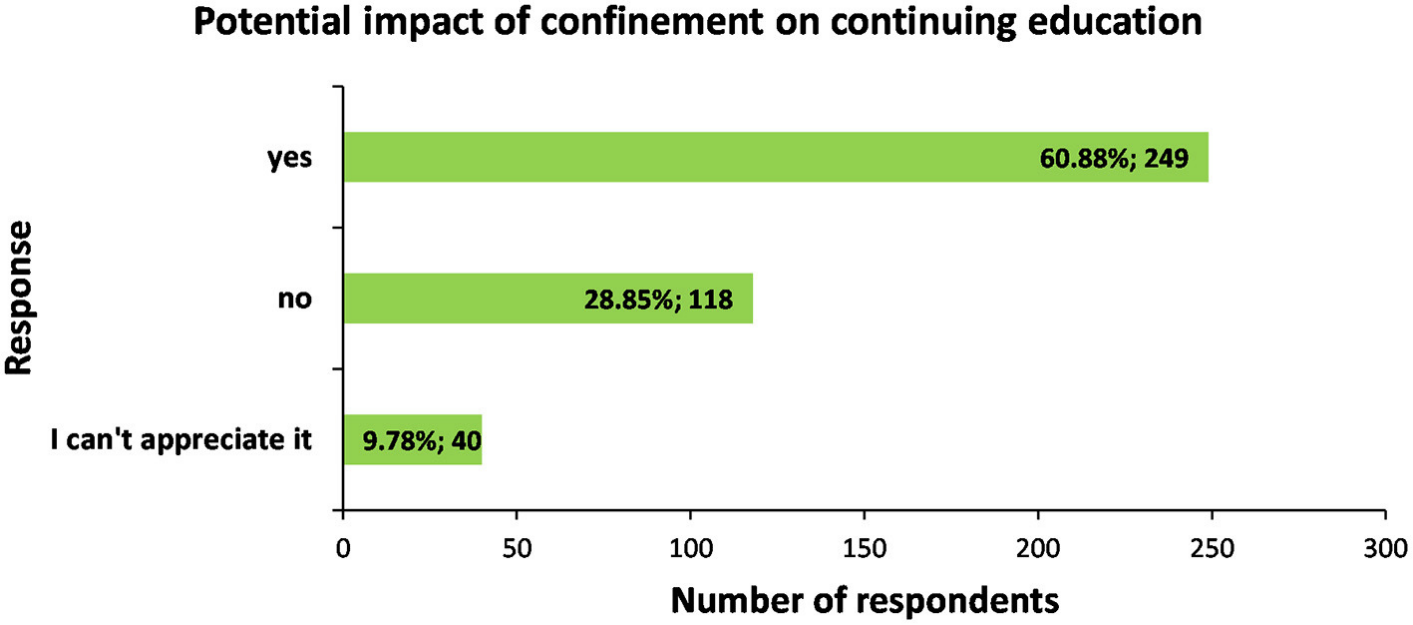
Distribution of respondents (*n* = 407) with respect to their perception about the potential impact of confinement on their continuing education.

Almost two-thirds of the respondents (60.88%; *n* = 249) considered that their continuing education has been affected by the COVID-19 pandemic. For 28.85% (*n* = 118) this was not the case and 9.78% (*n* = 40) were unsure, while 2 people (0.49%) skipped the answer (Figure 3).

Half of the respondents (50.12%; *n* = 205) took more online courses during confinement compared to the period before the COVID-19 pandemic. Nevertheless, 41.08% (*n* = 168) did not and 8.31% (*n* = 34) did not know or were not sure (2 skipped answers; 0.49%).

70.9% (*n* = 290) considered that an on-site event (such as an annual congress) would be epidemiologically risky (due to the risk of infection with SARS-CoV-2), but 13.69% (*n* = 56) did not, and 14.67% (*n* = 60) were not sure (3 skipped answers; 0.73%).

Considering the risk of infection with SARS-CoV-2, 43.28% (*n* = 177) did not want to attend an onsite continuing education event (e.g., an international congress in the country or abroad) within the next 6 months. However, 31.05% (n = 127) thought the probability of choosing to attend was small (under 25%), 13.2% (*n* = 54) appreciated the chances of attendance as medium, 5.62% (*n* = 23) thought the chances were high (over 75%); and 6.11% (*n* = 25) answered they would definitively participate to an onsite event (3 skipped answers; 0.73%).

Regarding the potential educational and professional impact of an online vs. an onsite event, 17.6% (*n* = 72) thought that both types of events have the same educational impact, 6.11% (*n* = 25) considered an online event superior to an onsite event, whereas 42.3% (*n* = 173) considered an online event to be inferior to an onsite event, and 33.5% (*n* = 137) thought that the two types of events cannot be compared, both being necessary (2 skipped answers; 0.49%).

In the free-text comment box, only 40 (9.78%) respondents addressed comments and suggestions, such as positive appreciations, online course requests, mentioning of struggles in communication and relationship between veterinarian and pet owners, the relationship issues between veterinary health authorities and veterinarians, or interest regarding the outcome of the survey.

## Discussion

This survey-based study provides a comprehensive view on Romanian veterinarians' opinion on COVID-19 lockdown impact on the profession. We hypothesized that the SARS-COV-2 virus outbreak and lockdown had significant consequences on well-being, health, and socio-economic aspects of veterinary professionals. Overall, the results of the study confirm that Romanian veterinarians were affected by the COVID-19 pandemic and lockdown measures on every studied aspect. Little has been published on this current topic with most focusing on animal health and well-being rather than veterinary staff (9, 11–14). One study, together with two letters to the editor of the same authors, revealed the impact of COVID-19 pandemic on American veterinary emergency professionals (15–17) as well as one comment to the editor referring to the Indochina region (18). Quain et al. (19) looked at the ethical challenges veterinary professionals had to experience during this unprecedented major public health burden and evaluated measures regarding biosecurity, client financial limitations, patient welfare, working conditions, and client relations, while Mahdy (20) evaluated the influence COVID-19 pandemic on veterinary student academic performance. Another two studies (21, 22) focused on equine practice and mental health of veterinary professionals during the pandemic. However, none looked at the effects of strict lockdown measures on the veterinary profession. Our results show that the impact of lockdown lies directly on four main categories of importance on veterinarian professional's life—human resource, activity management, relationship between veterinarian and authorities, and continuing education.

In the U.S. survey study involving 50 small animal emergency veterinary hospitals, 26% of the respondents were hospital directors and 42% had a leadership position (15), whereas in our case, most respondents were practicing veterinarians of all types, of which about half were owners or managers of mostly small veterinary clinics (under 5 employees), or employees in veterinary practice or industry (almost half), and only a small percentage of academic staff.

Few veterinarians in Romania saw reductions in staff/ unemployment/resignation; a more consistent percentage (36.19%) experienced decreased revenues in income, which is in contrast to less optimistic situations concerning other types of small businesses in other parts of the world, where closures and layoffs were much more frequent (23). The vast majority (over 80%) of the respondents were worried about various degrees of exposure to infection at work and felt that their professional environment was more stressful than usual, similar to a survey carried out by the British Veterinary Association in 2020 (24). This is concerning due to shortage of personal protective equipment during lockdown and is similar to the perception of many other medical professionals (15, 19, 25, 26).

However, during lockdown, few respondents were positive for SARS-CoV-2 or even tested at all, and only 15.4% knew someone who was tested positive. Similarly, Wayne and Rozanski reported at least one positive employee per hospital in the majority of responding emergency hospitals (15, 17).

The same authors yielded partially similar results with our study in terms of activity management (15). Most of their respondents reported significant changes to the hospital operation process, with 18% allowing emergencies only. For our surveyed veterinarians, working hours were reduced in more than half of workplaces and their caseload dropped. 20.29% saw an increase in caseload but almost half of the respondents saw similar types of cases compared to pre-lockdown, with also 18% of veterinarians receiving only emergency cases and an almost equal number receiving scheduling appointments only. This trend was also reported in human medicine, where lockdown has changed both the type and volume of caseload in many hospitals and medical centers (27–30).

While 9 in 10 respondents implemented at least some of the FECAVA/FVE guidelines (most frequently—wearing appropriate PPE by all staff, increased hand washing, maintenance of social distance at 2 meters from each other, and regular disinfection of all surfaces touched by both employees and clients), the least implemented measures were working from home, restriction of cases seen in the clinic (for emergencies only), restriction of owner access to the examination room and elimination of non-essential visits, in complete contrast to the American and U.K. population (15, 24, 31) where owner access was restricted in most hospitals with very few allowing access with restrictions, or no access at all. However, all over the world, most workplaces encountered deficiencies in the supply of veterinary pharmaceuticals and medical supplies during confinement.

Most respondents had to answer owner questions regarding SARS-CoV-2/COVID-19 transmission to and from pets, but only a few had to examine suspect cases or even animals that belonged to COVID-19 positive owners. Almost half of the respondents had to travel to the owner's home to examine pets (due to strict lockdown measures of not being allowed to travel for non-essential matters) and about a third were stopped at least once by the police in order to check for paperwork. Less than 2% of veterinarians were fined during these travels.

On a more positive note, the part of the survey dedicated to continuing education revealed that almost half of the responding vets attended more online courses than before lockdown. Almost two-thirds thought that on-site events would be too risky in the nearby future (6 months) and excluded the possibility of attending such an event. However, 40% considered the educational impact of online events inferior to on-site events, whereas <10% thought the exact opposite. These results mirror the opinion of veterinary students surveyed by Mahdy (20) who appreciated the flexibility of this type of learning but saw limited clinical applicability of virtual education. The topic of online continuing education resonates with the difficulties reported in human medical sciences, where the use of online platforms has proven invaluable during lockdown measures (32, 33) but also an opportunity that might shape the face of CE in the following years as well (34) as in-person meetings and events have been canceled for over a year and strict social distancing rules according to WHO recommendations have been implemented in most medical facilities (35), including veterinary ones, according to our surveyed veterinarians.

### Limitations of the Study

There was a rather limited number of respondents, especially from some specific groups (e.g., veterinary technicians, or academia). Having a larger number of participants could provide enhanced accuracy of the assessment, reducing the risk of under- or overestimation of the true COVID-19 pandemic impact. However, in Romania, there is a very low number of veterinary technicians compared to most of Europe. In addition, distribution of respondents relative to working place and age was considered adequate, based on the demographic data obtained. Since all authors of this study are working in academia, there was a unanimous opinion that COVID-19 pandemic had an immediate significant impact on this domain in a completely different manner, whose evaluation and analysis were beyond the scope of this study. Last but not least, another possibility is that many people were reluctant to answer, being more concerned about the first wave pandemic real-life challenges and consequences.

At present, Romania has no official statistics published regarding the number of veterinarians and technicians, and consequently, no response rate could be estimated.

It is worth noting that by running the survey after lockdown, for a period of almost 2 months, the responses might have changed in time accordingly, and that the timing of submission might have had an effect on the obtained data, such as evaluation of risk exposure or PPE shortage problems. However, from the total of 409 individuals who completed the survey, 90% of the respondents did so within the first 4 weeks.

Possible sources of bias associated with this research: voluntary surveys are subject to selection bias; responses may be subject to recall bias.

## Conclusion

Although the state of emergency declared for addressing COVID-19 illness outbreak lasted 60 days in Romania, it had a significant immediate impact on several aspects of the veterinary profession, with negative repercussions on human resource, activity management, relationship between veterinarian and authorities, and continuing education. Further studies are necessary to assess its potential long-term consequences as the COVID-19 pandemic has impacted veterinary and human healthcare in a possibly permanent way. Even at the time of writing this paper, several lockdowns are still implemented all over the world and might still be needed in the future until herd immunity can be reached through vaccination and natural immunity to SARS-CoV-2.

## Data Availability Statement

The raw data supporting the conclusions of this article will be made available by the authors, without undue reservation.

## Ethics Statement

The studies involving human participants were reviewed and approved by UASVM Cluj-Napoca Local Bioethics Committee (CBE protocol no. 265). Written informed consent for participation was not required for this study in accordance with the national legislation and the institutional requirements.

## Author Contributions

All authors listed have made a substantial, direct and intellectual contribution to the work, and approved it for publication.

## Funding

The publication was partially supported by funds from the Institutional Development Fund (IDF no. 0013) granted by the Romanian Ministry of Research and Innovation and awarded to AM and CM. The funder had no role in the conceptualization, study design, data collection and analysis, decision to publish, or preparation of the manuscript.

## Conflict of Interest

The authors declare that the research was conducted in the absence of any commercial or financial relationships that could be construed as a potential conflict of interest.

## Publisher's Note

All claims expressed in this article are solely those of the authors and do not necessarily represent those of their affiliated organizations, or those of the publisher, the editors and the reviewers. Any product that may be evaluated in this article, or claim that may be made by its manufacturer, is not guaranteed or endorsed by the publisher.
