# Supplementary material for: The Impact of COVID-19 Pandemic During Lockdown on the Veterinary Profession in Romania: A Questionnaire-Based Survey
Source: Front Vet Sci. 2021 Nov 10;8:737914. doi: 10.3389/fvets.2021.737914 (PMC8631325; doi:10.3389/fvets.2021.737914)
Supplement: Supplementary file 1 [file Data_Sheet_1.PDF]

Supplementary Material

Online questionnaire on

The Impact of COVID-19 Pandemic During Lockdown on the Veterinary Profession in Romania: a Questionnaire-based Survey

Impactul Pandemiei Covid-19 în Timpul Stării de Urgență asupra Profesiei de Medic Veterinar în Romania

ÎNTREBĂRI

RESURSA UMANĂ

1. În ce categorie profesională vă încadrați?
medic veterinar
medic veterinar primar
tehnician/asistent veterinar
manager clinică, medic veterinar
cadru didactic, specializare medicină veterinară

2. În ce rol vă încadrați?
angajat
proprietar/manager clinică
cadru didactic
angajat în unitate de asistență sanitar-veterinară

3. Unde vă desfășurați activitatea profesională?
cabinet veterinar privat
clinică veterinară privată
spital veterinar privat
clinică/spital universitar(ă)
farmacie veterinară/firmă distribuție farmaceutică
circumscripție sanitar veterinară
biobază
siguranța alimentelor și sănătate publică
structură de stat (ex. dsv, ansvsa, etc.)
altele (vă rugăm menționați ....)

4. De cât timp profesați?
0-5 ani
6-10 ani

11-20 ani
peste 20 ani

5. **Din câte persoane (medici veterinari, asistenți/tehnicieni veterinari, personal recepție, etc.) este formată echipa în care vă desfășurați activitatea profesională?**

1-5 angajați
5-10 angajați
10-20
peste 20

6. **Care a fost modalitatea de comunicare preferată la locul de muncă, pe durata stării de urgență?**

discuții scurte, cu păstrarea unei distanțe cel puțin 1,5 metri
mesaje scrise
mesaje electronice/ poșta electronică
alte metode (vă rugăm să le specificați) ....

7. **Au existat reduceri de personal în cadrul structurii unde vă desfășurați activitatea profesională în timpul și respectiv după încetarea stării de urgență?**

da
nu
nu știu

8. **Au existat situații de șomaj tehnic în cadrul structurii unde vă desfășurați activitatea profesională în timpul și respectiv după încetarea stării de urgență?**

- da
- nu
- nu știu

9. **După revenirea la activitatea zilnică uzuală, respectiv încetarea stării de urgență, au existat demisii în cadrul structurii unde vă desfășurați activitatea profesională?**

da
nu
nu știu
nu se aplică

10. **Veniturile provenite din salariile proprii au suferit modificări în perioada menționată?**

da, au crescut
da, au scăzut
nu, au rămas constante

11. **În timpul stării de urgență, la locul de muncă, v-ați simțit:**

în siguranță
expus/ă unui risc ridicat de contaminare
expus/ă unui risc mediu de contaminare
expus/ă unui risc mic de contaminare

12. **Sunteți de acord cu următoarea afirmație? În timpul stării de urgență activitatea mea profesională a fost mai stresantă decât de obicei.**

total de acord
parțial de acord
dezacord parțial
dezacord total
nu știu/nu îmi dau seama

13. **În cercul dvs. de cunoscuți au existat persoane testate pozitiv pentru SARS-CoV-2?**

da, o persoană
da, mai multe persoane
nu, nu cunosc

14. **Ați fost testat(ă) pentru SARS-CoV-2?**

nu, nu am fost testat
da, am fost testat, cu rezultat negativ
da, am fost testat, cu rezultat pozitiv

15. **În urma testării pozitive pentru SARS-CoV-2, ați fost spitalizat(ă)?**

- da
- nu
- neaplicabil

16. **În timpul stării de urgență ați avut în echipa în care vă desfășurați activitatea profesională persoane din categorii vulnerabile (femei însărcinate, bolnavi cronici, personal peste 65 de ani)?**

- da
- nu
- nu știu

17. **Considerați că în timpul stării de urgență ați avut parte și de oportunități ca de exemplu?**

mai mult timp liber
învățarea unor lucruri noi
schimbări de obiceiuri sau rutină zilnică
stabilirea/reluarea unor legături sociale
acțiuni de ajutor social
reducerea poluării sau a traficului (rutier)

conștientizarea rolului/limitelor ființei umane

GESTIONAREA CAZURILOR/ACTIVITĂȚII

18. La locul dvs. de muncă în timpul stării de urgență s-a înregistrat o schimbare a programului zilnic?

nu
da, o reducere a programului de lucru cu publicul
da, o extindere a programului de lucru cu publicul
da, am închis temporar
da, am închis definitiv

19. La locul dvs. de muncă s-a înregistrat o schimbare în volumul de cazuri gestionate, în timpul stării de urgență?

da, am avut mai multe cazuri
da, am avut mai puține cazuri
nu, volumul de cazuri a fost nemodificat
nu știu
neaplicabil

20. La locul dvs. de muncă s-a înregistrat o schimbare a tipului de cazuri gestionate, în perioada de stare de urgență?

nu
da, am primit exclusiv urgențe
da, am primit exclusiv cazurile programate
da, am redirecționat toate cazurile
neaplicabil

21. Considerați că restricțiile impuse în timpul stării de urgență au influențat decizia proprietarului de a se prezenta la medic cu pacientul?

- nu
- da
- nu știu/nu îmi dau seama
- neaplicabil

22. Care au fost măsurile implementate la locul dumneavoastră de muncă în timpul perioadei de urgență:

- obligativitatea purtării echipamentului de protecție adecvat (ex. măști, mănuși)
- menținerea unei distanțe de siguranță de 1.5-2 metri între persoane
- furnizarea unui dezinfectant de mâini plasat la vedere în zona de acces/ieșire
- curățarea și dezinfectarea zonelor atinse de către mai multe persoane (ex. mânerele ușilor)

- permiterea efectuării activității profesionale de la domiciliu pentru personalul angajat (ex. secretar)
- scurtarea programului de lucru
- plata cu cardul (contactless)
- restricționarea activității clinice exclusiv pentru cazurile de urgență
- programarea exclusiv telefonică/online a cazuisticii
- informarea verbală a proprietarilor de animale despre măsurile noi de protecție
- informarea prin documente printate, plasate la vedere a proprietarilor de animale despre măsurile noi de protecție
- restricționarea accesului la un singur proprietar per animal în sala de consult și sala de așteptare
- spălarea pe mâini după fiecare interacțiune cu un animal și respectiv proprietar
- împărțirea echipei în două (sau 3) grupuri, cu evitarea contactului fizic între acestea
- eliminarea lucrurilor care pot fi atinse de către multiple persoane din sala de așteptare
- eliminarea orelor de vizită și eliminarea vizitelor neesențiale (ex. a reprezentanților medicali, etc.) (multiple choice)

23. **În timpul stării de urgență, în echipa în care vă desfășurați activitatea profesională au fost luate măsuri speciale pentru persoanele angajate din categorii vulnerabile (femei însărcinate, bolnavi cronici, personal peste 65 de ani)?**

da (va rugăm să specificați aceste măsuri) ....
nu
nu știu
nu am avut colegi persoane din categorii vulnerabile

24. **La locul dvs. de muncă au fost instituite alte modalități alternative de furnizare a serviciilor de asistență medicală și diagnostic, în ideea menținerii distanțării sociale? (ex. telemedicina, consultație fără prezența proprietarului, triaj telefonic)**

da
nu
nu știu/nu îmi amintesc
neaplicabil

25. **La locul dvs. de muncă au existat deficiențe în aprovizionarea cu produse farmaceutice veterinare, medicamente de uz veterinar și consumabile medicale în timpul stării de urgență?**

da, deficiențe majore
da, deficiențe minore
nu am avut astfel de deficiențe
nu știu/nu îmi amintesc

neaplicabil

26. **La locul dvs. de muncă au existat solicitări de sprijin cu produse medicamentoase, consumabile medicale sau aparatură medicală (ex. monitoare funcții vitale, ventilatoare), din partea unor furnizori de servicii medicale umane, în timpul stării de urgență?**

da
nu
nu știu/nu îmi amintesc
neaplicabil

27. **Pentru desfășurarea în bune condiții a activității profesionale în timpul stării de urgenţă a fost necesară modificarea plafonului de tarifare pentru serviciile medical-veterinare prestate la locul dvs. de muncă?**

da, a fost necesară, au scăzut
da, a fost necesară, au crescut
nu, nu a fost necesară
neaplicabil

28. **V-au fost solicitate de către către proprietarii de animale informații privind posibilitatea transmiterii SARS-CoV-2 de la animale la om sau viceversa?**

da
nu
nu îmi amintesc

29. **Ați fost solicitat(ă) să consultați un animal suspect de SARS-CoV-2, sau să efectuați teste pentru un astfel de diagnostic?**

da
nu
nu îmi amintesc
neaplicabil

30. **Ați fost solicitat(ă) să consultați un animal aparținând unui proprietar suspect/ confirmat pozitiv pentru SARS-CoV-2?**

da
nu
nu îmi amintesc
neaplicabil

31. **Ați fost solicitat(ă) de către un proprietar de animale să efectuați la un animal clinic sănătos o vaccinare cu un vaccin comercial canin împotriva coronavirusului enteric, pentru o eventuală cross-protecție împotriva SARS-CoV-2)?**

da
nu

nu îmi amintesc
neaplicabil

RELAȚIA DINTRE MEDICUL VETERINAR ȘI ORGANELE DE CONTROL

32. În timpul stării de urgență ați fost nevoit(ă) sa efectuați deplasări în teren sau la domiciliul clientului pentru consultații și manopere medical-veterinare?
da, foarte des
da, însă ocazional (de 1-2 ori pe săptămână)
nu mi s-a solicitat niciodată
nu, am refuzat să efectuez astfel de servicii
neaplicabil

33. În timpul deplasării în teren/la domiciliul clientului în interes medical-veterinar ați fost opriți de către organele de control în scopul verificării respectării interdicţiilor impuse pe timpul stării de urgenţă?
da, mai des de o dată pe săptămână
da, ocazional (1 dată pe săptămână)
nu, niciodată
neaplicabil

34. În timpul deplasării în teren/la domiciliul clientului în interes medical-veterinar vi s-a emis vreodată de către organele de control un proces verbal de contravenție pe durata stării de urgenţă?
- da
- nu
- neaplicabil

EDUCAȚIA CONTINUĂ

35. Considerați că educația dvs. continuă a fost afectată de pandemia COVID-19?
- da
- nu
- nu pot aprecia

36. Ați făcut în perioada de stare de urgență mai multe cursuri online decât în perioada de dinainte de pandemia COVID-19?
- da
- nu
- nu pot aprecia, nu îmi dau seama

37. Având în vedere situația actuală și implicit riscul de infectare cu SARS-CoV-2, considerați că un eveniment on-site (ca de exemplu un congres anual) ar fi riscant din punct de vedere epidemiologic?

- da
- nu
- nu pot aprecia, nu îmi dau seama

38. Având în vedere situația actuală și implicit riscul de infectare cu SARS-CoV-2, care este probabilitatea de a participa la un eveniment de educație continuă on-site (ca de exemplu un congres internațional, în țară sau străinătate) în următoarele 6 luni?

Nu voi participa
Mică, sub 25%
Medie, aproximativ 50%
Mare, peste 75%
Voi participa 100%

39. Care ar fi din punctul dvs. de vedere impactul potențial, educațional și profesional al unui eveniment online versus un eveniment clasic (on-site)?

are același impact educațional și profesional
un eveniment online este **superior** celui on-site
un eveniment online este **inferior** celui on-site
cele două tipuri de evenimente nu se pot compara, ambele fiind necesare

40. În cazul în care doriți să adăugați un comentariu, vă rugăm să utilizați caseta de text.

Vă mulțumim încă odată pentru timpul acordat.
